# Supplementary material for: Chemical Modifications Influence the Number of siRNA Molecules Adsorbed on Gold Nanoparticles and the Efficiency of Downregulation of a Target Protein
Source: Nanomaterials (Basel). 2022 Dec 14;12(24):4450. doi: 10.3390/nano12244450 (PMC9781748; doi:10.3390/nano12244450)
Supplement: Supplementary file 1 [file nanomaterials-12-04450-s001.zip › nanomaterials-2056321-supplementary.pdf]

nanomaterials

MDPI

Supplementary Materials

Chemical Modifications Influence the Number of siRNA Molecules Adsorbed on Gold Nanoparticles and the Efficiency of Downregulation of a Target Protein

Anna V. Epanchintseva, Julia E. Poletaeva, Anton S. Dome, Ilya S. Dovydenko *, Inna A. Pyshnaya and Elena I. Ryabchikova *

Institute of Chemical Biology and Fundamental Medicine SB RAS, Novosibirsk 630090, Russia
* Correspondence: dovydenko_il@niboch.nsc.ru (I.S.D.); lenryab@yandex.ru (E.I.R.); Tel.: +7-(383)-363-5163 (I.S.D. & E.I.R.)

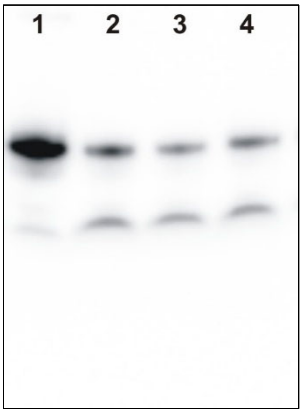

Figure S1. Assessment of MLNC resistance to nucleases by polyacrylamide gel electrophoresis. 1-control siRNA sample, 2-4 – siRNA samples after 4 h incubation in 10% FBS, experiment was carried out in triplicate.

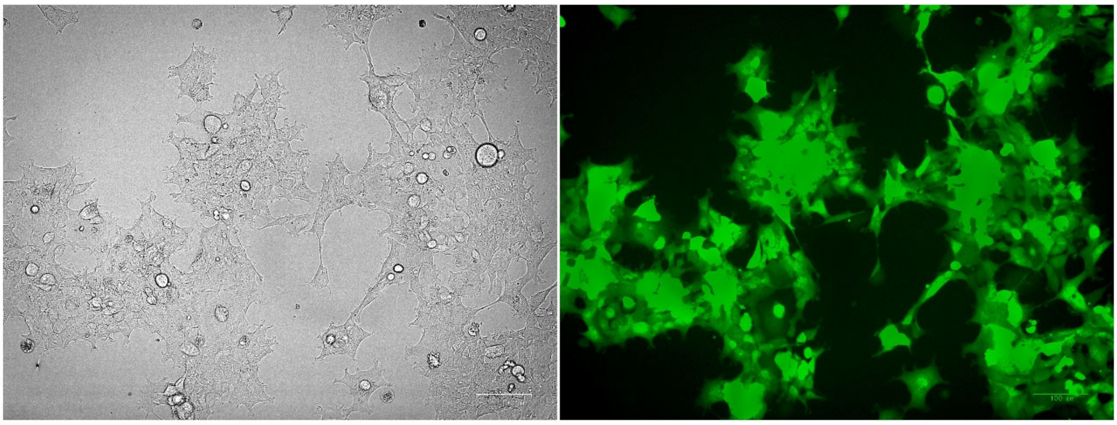

Figure S2. EGFP-expressing HEK293FT cells. The image of the cells on well surface in phase-contrast mode (left) and in ultraviolet light (right). The intense green fluorescence of EGFP protein in the cells is observed.

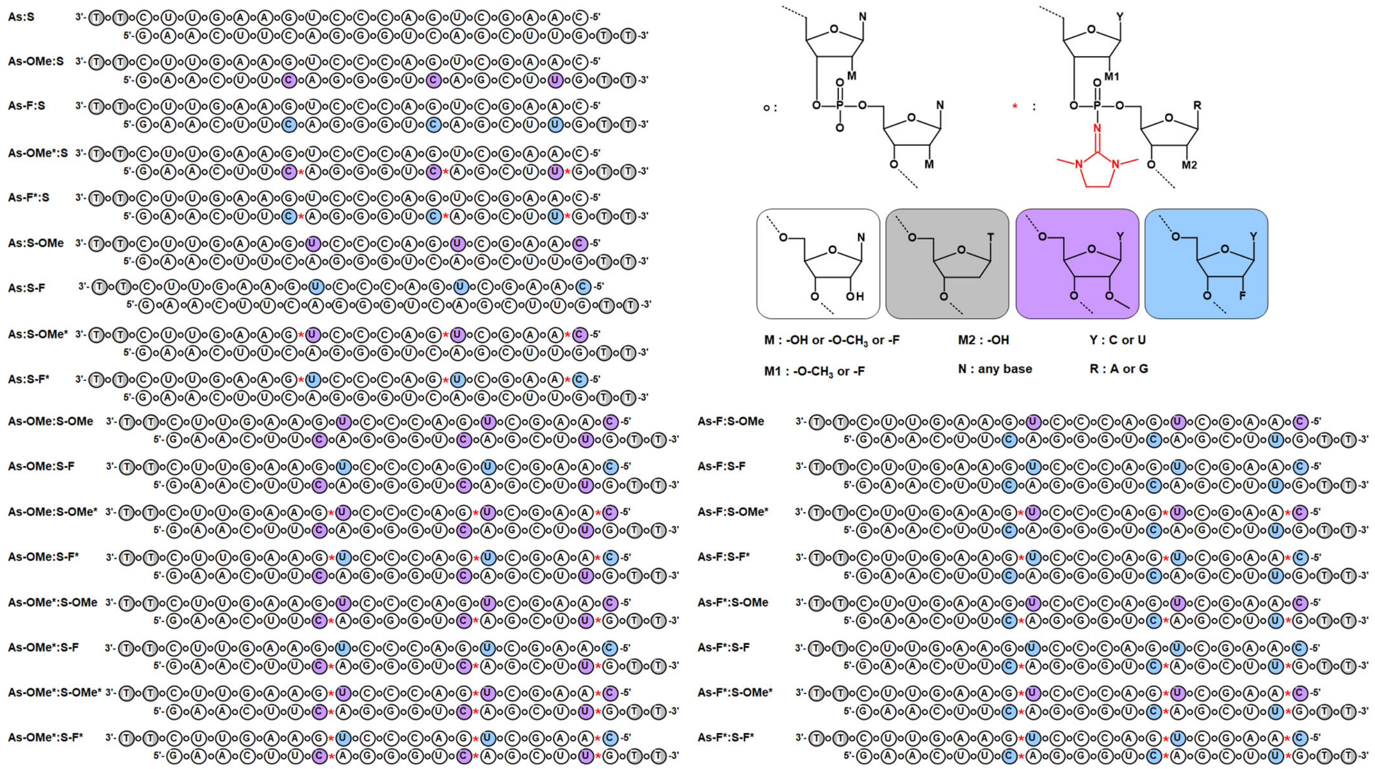

Figure S3. The library of obtained duplexes: the unmodified siRNA and siRNAs bearing combined modifications PG/2′-OMe or PG/2′-F or containing only 2′-OMe and 2′-F substitutions. **As**: the antisense strand in an siRNA, **S**: the sense strand; the asterisk represents a PG-modified phosphate group. Adapted from [25].
